# The Association between Walking and Perceived Environment in Chinese Community Residents: A Cross-Sectional Study

**DOI:** 10.1371/journal.pone.0090078

**Published:** 2014-02-27

**Authors:** Yingnan Jia, Tricia Usagawa, Hua Fu

**Affiliations:** 1 School of Public Health, Key Lab of Public Health Safety of the Ministry of Education, Fudan University, Shanghai, China; 2 Health Communication Institute, Fudan University, Shanghai, China; 3 Office of Public Health Studies, University of Hawaii, Honolulu, Hawaii, United States of America; University of Bologna, Italy

## Abstract

**Background:**

The neighborhood environment, as a determinant of walking, has been assessed in several developed countries. However, few studies have investigated these associations in Chinese populations.

**Objective:**

To examine the association between the perceived neighborhood environment and walking for recreation or transportation purposes among Chinese community residents.

**Methods:**

We used a multi-stage stratified random sampling design to conduct a cross-sectional study of 1528 Chinese adults in Shanghai. Environmental and walking variables were assessed using a revised Abbreviated Neighborhood Environment Walkability Scale for Chinese subjects and a long version of International Physical Activity Questionnaire. Self-reported demographic variables including gender, age, employment status, and location of community were also collected. Multiple logistic regression analysis was applied to examine the association between the neighborhood environment and walking.

**Results:**

Based on the results of IPAQ, 13.7% of the overall subjects were physical inactive, which was considered to be lowly active. For all participants, accessibility to services was significantly associated with walking for both recreation and transportation (odds ratio = 1.062, 95% confidence interval: 1.016, 1.110; odds ratio = 1.053; 95% confidence interval: 1.008, 1.100, respectively). In males, accessibility to services was significantly associated both with walking for recreation and walking for transportation. However, a significantly negative association was found between the neighborhood surroundings and walking for recreation. In contrast, females who perceived good traffic safety tended to walk for recreation. Data also revealed a difference between working and retired individuals. Among working participants, perceived environmental variables were not significantly associated with walking for recreation and transportation.

**Conclusions:**

The association between neighborhood environment and walking varied depending on the reason for walking and the characteristics of the participants. Our findings suggest that interventions to promote walking in community residents should include improving the neighborhood environment, particularly accessibility to services such as building more stores, parks, and public transit facilities.

## Introduction

Regular physical activity is an important contributor to health, and reduces the risk of many chronic diseases [1]–[3]. With the economic development of China, the percentage of people taking part in moderate and vigorous physical activity during their leisure time is generally <20% [4]–[6]. Moreover, a longitudinal study reported that the decline in physical activity levels among Chinese adults was strongly associated with the increased availability of higher educational institutions, housing infrastructure, sanitation improvements, and the economic wellbeing of the community in which people function [6]. Therefore, how to promote physical activity is an important public health question [6]. Most research into the possible physical activity determinants for intervention has focused on individual demographics and psychosocial factors [7]. More recently, some studies suggested that associations between the built environment and physical activity were in the expected direction or null [8]–[14]. Land use diversity, connectivity, accessibility to services, aesthetics, safety, population density, and overall neighborhood design were important environmental determinants for physical activity [14]. Unlike other types of physical activity such as walking for leisure, the built environment was more likely to be associated with walking for transportation [14]. In addition, several studies reported differences between males and females [10], [15].

Although several studies have been performed in developed countries, few studies investigating the association between the built environment and physical activity have been conducted in developing countries. There are significant differences in the living conditions, population density, and working demands between developed and developing countries due to different economic and cultural factors. Therefore, the residents’ cognition and attributes for environmental needs would differ as well as physical activity behaviors. In urban China, for example, the per capita living space is 31.6 m^2^ with a population density of 2209 people per km^2^. The population density in Shanghai is even higher, with 3630 people per km^2^ [16]. Compared with western countries, retired people used to be the most physically active population in China [5]. These differences might lead to different associations between the environment and physical activity. Results of a study conducted in Hong Kong showed a general lack of association between overall walking and perceived environmental attributes [17]. An additional study conducted in Taiwan demonstrated that accessibility to facilities had a significant impact on the physical activity of children [18]. In Mainland China, related studies have focused mainly on the development and assessment of physical activity relative scales such as the Chinese version of the International Physical Activity Questionnaires, Neighborhood Physical Activity Questionnaire (NPAQ), and a Chinese version of Abbreviated Neighborhood Environment Walkability Scale (ANEWS) [19]–[21]. The present study was the first to examine the association between neighborhood environment and walking in adults based on community in Mainland China. Because walking is the most common recreational physical activity in the Chinese population [17], we selected walking as the main outcome in the present study. However, the environmental variables associated with walking might differ depending on the purpose of walking and the characteristics of the participants [9], [22]. Therefore, the aim of the current study was to examine the environmental determinants that could be targeted as interventions to promote physical activity. Interventions in a built environment might be more effective in China due to the high population density and centralized residences, where many people live in high-rise buildings. In addition, compared with physical activity interventions for individuals, changes to the environment are believed to have broader and longer-term impacts [23]. The evidence from China could support the results of studies conducted in other countries, and may introduce novel findings regarding associations between the environment and physical activity.

## Methods

### 2.1 Study Population and Sampling

The subjects were selected with two-stages of sampling for the study. During the first stage, 17 different neighborhoods were selected randomly in the Minhang district of Shanghai, China. The population density of these neighborhoods is similar, since all consisted of apartment buildings. However, there were differences in the environmental features of the 17 neighborhoods (such as accessibility to services, aesthetics, and facilities) due to the different economic conditions. In the second selection stage, 1800 participants aged 15−75 years were identified randomly determined after stratifying by gender and age groups.

### 2.2 Ethical Permission

Written Informed Consent Statement Forms were obtained from the respondents. The right to withdraw and autonomy of the respondents were explained. The study received ethical permission from the ethics committee of School of Public Health of Fudan University, China 51 (IRB00002408&FWA00002399).

### 2.3 Data Collection

A questionnaire regarding physical activity and the neighborhood environment was administered to the participants. The questionnaire included four parts: (1) demographic characteristics, (2) a long version of International Physical Activity Questionnaire (IPAQ) in Chinese, (3) Chinese Neighborhood Environment Walkability Scale (CNEWS), which was modified based on ANEWS, and (4) three questions assessing knowledge of physical activity.

### 2.4 Demographics and other Variables

Self-reported demographic variables included gender, age, education levels, employment status, body height and weight, and marital status. Other variables, including the location of the community and hypertension, were also collected.

### 2.5 Measurement of Physical Activity

The self-reported data based on the Chinese version of long IPAQ were summarized into four physical activity domains (occupational, transport, household, and leisure related physical activities) and the estimated total time spent in each domains and the total time spent sedentary per week was reported [24], [25]. The IPAQ survey and scoring protocols (available online at: http://www.ipaq.ki.se) were adopted for this study. There were three levels of physical activity used to classify populations: low, moderate, and high. The two criteria for classification as ‘high’ were (a) vigorous-intensity activity on at least 3 days, achieving a minimum total physical activity of at least 1500 Metabolism Equivalents (METs) -minutes/week OR (b) 7 or more days of any combination of walking, moderate-intensity, or vigorous-intensity activities achieving a minimum total physical activity of at least 3000 MET-minutes/week. ‘Moderate’ activity was classified as: (a) 3 or more days of vigorous-intensity activity of at least 20 minutes per day OR (b) five or more days of moderate-intensity activity and/or walking for at least 30 minutes per day OR (c) five or more days of any combination of walking, moderate-intensity, or vigorous intensity activities achieving a minimum total physical activity of at least 600 MET-minutes/week. Individuals who did not meet the criteria for ‘moderate’ or ‘high’ were considered to have a ‘low’ physical activity level.

### 2.6 Assessment of Walking

Self-reported physical activity in the last seven days was collected using the IPAQ. The frequency (number of days) and duration (hours and minutes per day) of physical activity in the different domains (work, transportation, recreation, and household) were assessed. Items related to transportation walking were used to assess the number of days during the previous week spent walking at least 10 min from place to place, and the typical minutes per day. The time spent leisure walking was assessed using similarly structured items.

### 2.7 Neighborhood Walkability Measurement

Because of the environmental and cultural differences between western countries and China, we developed a modified version of ANEWS for Chinese residents (CNEWS). We predetermined three communities with good, modest, and poor walkable environments separately based on prior observation. Twenty adults were then selected from each of these three neighborhoods. Each individual was interviewed face-to-face using the items in the modified version of ANEWS. All the residents were asked to sort the items based on their perception of the contribution of the environment to physical activity. A score would then be given to each item based on the sorting from the residents. Three items (trees, sanitation, and crime safety) were the most important, and so were weighted with double scores. An intercept convenient sample of 360 community residents (>20 years old) from two neighborhoods with different walkable environments were interviewed. The results showed that the CNEWS for urban community residents had good reliability and validity, and could be used as an effective tool for assessing the walkable environment of urban communities in China [21].

A neighborhood-level walkability index with four parts (accessibility to services, neighborhood surroundings/aesthetics, traffic safety, and crime safety variables) was calculated using the CNEWS, which had showed good reliability previously (intra-class range from 0.802 to 0.947) [21]. Access to services included four items (stores, parks, bus/train stops, and other places). Neighborhood surroundings/aesthetics included seven items (trees, sanitation, lighting, condition of roads, natural sights, interesting things, and places for walking). Traffic safety included three items (traffic accidents, obstruction of traffic, and speed of traffic). Crime safety included three items (total crime rate, crime rate during the day, and crime rate at night). Each item was scored as: 1 = strongly disagree; 2 = somewhat disagree, 3 = unsure, 4 = somewhat agree, 5 = strongly agree. Therefore, a higher value indicated a more PA-friendly environment.

### 2.8 Analyses

Multiple logistic regression analysis was applied to examine the association between the neighborhood environment and walking. Walking for recreation and walking for transportation were used as dependent variables. The independent variables in the analysis were four perceived neighborhood environmental variables. For walking variables, the participants were classified into two groups using the median of 90 minutes per week: ≤90 min/week or >90 min/week. The scores for the four environmental variables, however, were used as continuous type variables. Odds ratios were adjusted by gender, age, location of the community, education levels, employment status, BMI, marital status, physical activity knowledge score, and hypertension. For all analyses, statistical significance was set at p<0.05. Statistical analyses were performed using the Statistical Package for Social Sciences (SPSS 20.0).

## Results

### 3.1 Participant Characteristics and Representation

Subjects (1800) from 17 neighborhoods were selected to participate in the study. Of these, 1528 participants (49.2% male and, 50.8% female) with complete data were used in the statistical analyses; the response rate was 84.9%. The demographics of the qualified participants are presented in Table 1. The age of the participants ranged from 15−75. The proportion of overweight participants (BMI≥24 kg/m^2^, the national criteria for being overweight in Mainland China) was 33.9%, 74.6% had been married, 32.7% had a college/university degree, and 45.3% were employed.

**Table 1:** Demographic characteristics of the participants.

	Overall		Males		Females	
	*n* = 1528		*n* = 752		*n* = 776	
	*n*	%	*n*	%	*n*	%
**Age (years)**						
** 15−24**	271	17.7	139	18.5	132	17.0
** 25−34**	271	17.7	131	17.4	140	18.0
** 35−44**	243	15.9	126	16.8	117	15.1
** 45−54**	261	17.1	127	16.9	134	17.3
** 55−64**	245	16.0	117	15.6	128	16.5
** 65−75**	237	15.5	112	14.9	125	16.1
**BMI**						
** ≤18.5**	139	9.1	70	9.3	69	8.9
** 18.5−24**	841	55.0	428	56.9	413	53.2
** 24−27**	354	23.2	159	21.1	195	25.1
** 27≤**	163	10.7	82	10.9	81	10.4
** Unanswered**	31	2.0	13	1.7	18	2.3
**Marital status**						
** Unmarried/Divorced/Widowed**	388	25.4	209	27.8	179	23.1
** Married**	1,140	74.6	543	72.2	597	76.9
**Education**						
** Junior high school**	568	37.2	247	32.8	321	41.4
** High School/technical secondary school**	457	29.9	235	31.3	222	28.6
** Junior college**	208	13.6	106	14.1	102	13.1
** Bachelor’s/Masters/Doctorate**	295	19.1	164	21.8	131	16.9
**Employment status**						
** Employed**	692	45.3	403	53.6	289	37.2
** Retired people**	445	29.1	175	23.3	270	34.8
** Student**	164	10.7	84	11.2	80	10.3
** Unemployed**	115	7.5	38	5.1	77	9.9
** Others**	112	7.3	52	6.9	60	7.7

### 3.2 Levels of Physical Activity

The distribution of physical activity levels is presented in Table 2. In all subjects, 50.9% and 35.5% had undertaken high and moderate levels of physical activities, respectively. The prevalence of low physical activity (physical inactivity) was 13.7%. When data were stratified by gender, 46.0% of males and 55.5% of females were classified into the high physical activity level group, whereas 16.0% of males and 11.5% of females had a low level of physical activity. The distribution of physical activity level also differed by age significantly.

**Table 2:** Physical activity level of participants.

Physicalactivitylevel	Total	High	Moderate	Low	χ^2^
	*n*	*n* (%)	*n* (%)	*n* (%)	
Overall	1528	777 (50.9)	542 (35.5)	209 (13.7)	/
Male	752	346 (46.0)	286 (38.0)	120 (16.0)	15.184**
Female	776	431 (55.5)	256 (33.0)	89 (11.5)	
15−34	542	218 (40.2)	217 (40.0)	107 (19.7)	65.192**
35−54	504	259 (51.4)	172 (34.1)	73 (14.5)	
55−75	482	300 (62.2)	153 (31.7)	29 (6.0)	
Working individuals	692	319 (46.1)	263 (38.0)	110 (15.9)	39.411**
Retired individuals	445	279 (62.7)	139 (31.2)	27 (6.1)	

*Significant at *P*<0.05,

**Significant at *P*<0.01.

### 3.3 Perceived Neighborhood Walkable Environments

Table 3 shows the mean scores and SDs for the four perceived environmental variables (accessibility to services, neighborhood surroundings/aesthetics, traffic safety, and crime safety). The participants were categorized by gender, age, employment status, and walking status. Of the perceived environmental variables, active walkers for recreation perceived significantly better accessibility to services than inactive walkers for recreation. Compared with inactive walkers for transportation, active walkers for transportation perceived significantly better accessibility to services and neighborhood surroundings/aesthetics. There were no significant differences between males and females or the different age groups in any perceived environmental variables. However, the scores of accessibility to services, crime safety, and total environmental score were significantly different between participants with different employment statuses. Working people and students rated significantly better accessibility to services, crime safety, and total environment scores than unemployed individuals. Retired people and students perceived better neighborhood surroundings than other participants, but this was not statistically significant.

**Table 3:** Results of perceived neighborhood walkable environmental measures.

	Access to services		Neighborhood surroundings/aesthetics		Traffic safety		Crime safety		Total environmental score	
	Mean±SD	*P* value	Mean±SD	*P* value	Mean±SD	*P* value	Mean±SD	*P* value	Mean±SD	*P* value
Active walkers for recreation	14.46±2.9	0.002	30.06±5.7	0.706	10.73±2.6	0.291	13.90±3.1	0.707	69.15±9.9	0.130
Inactive walkers for recreation	14.00±2.9		29.95±5.5		10.59±2.5		13.84±3.1		68.39±9.8	
Active walkers for transportation	14.40±2.9	0.008	29.74±5.8	0.043	10.55±2.5	0.068	13.85±3.2	0.753	68.54±10.2	0.352
Inactive walkers for transportation	14.00±2.9		30.32±5.4		10.79±2.6		13.90±3.0		69.01±9.4	
Male	14.22±2.84	0.929	30.01±5.71	0.978	10.62±2.65	0.634	13.91±3.10	0.658	68.76±9.87	0.952
Female	14.21±3.05		30.00±5.50		10.69±2.43		13.84±3.13		68.73±9.79	
15−29 yrs.	14.31±3.06	0.680	29.87±5.67	0.274	10.67±2.58	0.895	14.12±3.05	0.179	68.96±9.97	0.571
30−59 yrs.	14.16±2.81		29.88±5.43		10.67±2.46		13.77±3.09		68.49±9.60	
60−75 yrs.	14.24±3.11		30.43±5.87		10.60±2.68		13.81±3.23		69.07±10.15	
Employed	14.51±2.83	<0.001*	30.09±5.76	0.094	10.66±2.54	0.419	14.08±3.00	0.001*	69.34±9.96	0.001*
Retired	14.14±3.02		30.22±5.65		10.74±2.51		13.78±3.18		68.88±9.99	
Students	14.41±3.12		30.16±5.44		10.60±2.54		14.13±3.12		69.30±9.54	
Unemployed	13.36±3.15		29.83±5.14		10.24±2.64		13.54±3.36		66.97±9.40	
Others	13.30±2.50		28.62±4.92		10.78±2.53		12.88±3.12		65.57±8.43	

*Significant at *P*<0.05.

### 3.4 Results of Logistic Regression Analyses

Logistic regression analysis was employed to adjust for confounding factors that influence walking. Walking for recreation and transportation were used as the outcome variables. For walking variables, the participants were classified into two groups based on the median of 90 minutes per week: ≤90 min/week or >90 min/week. The independent variables used as continuous type variables included accessibility to services, neighborhood surroundings/aesthetics, traffic safety, crime safety, age, BMI, and physical activity knowledge score. The other independent variables used as categorical variables were gender, location of community, education level, employment status, marital status, and hypertension.

The result of logistic regression is presented in Table 4. Data revealed that for all participants, the score for accessibility to services was significantly associated with walking for recreation. As such, more participants walked for recreation and transportation when they perceived a better accessibility to services. The odds ratio was 1.062 with 95% confidence interval of 1.016 and 1.110 for walking for recreation, and 1.053 with 95% confidence interval of 1.008 and 1.100 for walking for transportation. After being stratified by gender and employment status, data revealed a gender-specific association between the environment and walking. In males, accessibility to services was significantly associated both with walking for recreation and for transportation. However, the neighborhood surroundings score was significantly negatively associated with walking for recreation. Among females, those who perceived good traffic safety tended to walk for recreation (odds ratio = 1.092; 95% confidence interval: 1.018, 1.171). There were also differences between working and retired individuals. Among working participants, perceived environmental variables were not significantly associated with walking for recreation or transportation. However, among retired individuals, those who perceived good accessibility to services were more likely to walk for transportation. The other environmental variables were not significantly associated with walking.

**Table 4:** Odds ratios for active walkers (all respondents, males, females, and working and retired individuals).

		Walking for Recreation (>90 min/week group, ≤90 min/weekgroup)		Walking for transportation(>90 min/week group, ≤90 min/week group)	
		OR (95%CI)	*P* value	OR (95%CI)	*P* value
All respondents (*n* = 1499)	Access to services	1.062 (1.016, 1.110)	0.008	1.053 (1.008, 1.100)	0.020
	Neighborhood surroundings/aesthetics	0.986 (0.961, 1.011)	0.271	0.975 (0.951, 1.000)	0.053
	Traffic safety	1.036 (0.989, 1.086)	0.139	0.973 (0.929, 1.019)	0.252
	Crime safety	1.000 (0.959, 1.043)	0.991	1.001 (0.960, 1.043)	0.972
Male (*n* = 740)	Access to services	1.130 (1.054, 1.210)	0.001	1.080 (1.012, 1.154)	0.021
	Neighborhood surroundings/aesthetics	0.963 (0.927, 0.999)	0.046	0.981 (0.946, 1.018)	0.315
	Traffic safety	0.989 (0.925, 1.057)	0.774	0.962 (0.901, 1.028)	0.251
	Crime safety	1.064 (0.998, 1.133)	0.056	0.982 (0.924, 1.044)	0.559
Female (*n* = 759)	Access to services	1.014 (0.953, 1.079)	0.655	1.029 (0.968, 1.093)	0.364
	Neighborhood surroundings/aesthetics	1.007 (0.971, 1.044)	0.723	0.970 (0.935, 1.006)	0.100
	Traffic safety	1.092 (1.018, 1.171)	0.014	0.979 (0.914, 1.049)	0.548
	Crime safety	0.951 (0.896, 1.009)	0.096	1.018 (0.959, 1.081)	0.559
Working people (*n* = 681)	Access to services	1.055 (0.987, 1.128)	0.114	1.046 (0.979, 1.117)	0.187
	Neighborhood surroundings/aesthetics	0.984 (0.948, 1.021)	0.383	0.971 (0.936, 1.007)	0.116
	Traffic safety	1.036 (0.967, 1.110)	0.313	0.952 (0.889, 1.021)	0.168
	Crime safety	1.019 (0.954, 1.088)	0.575	1.019 (0.955, 1.088)	0.562
Retired respondents (*n* = 441)	Access to services	1.056 (0.962, 1.160)	0.252	1.097 (1.001, 1.202)	0.048
	Neighborhood surroundings/aesthetics	0.995 (0.944, 1.047)	0.837	0.976 (0.926, 1.028)	0.359
	Traffic safety	1.009 (0.916, 1.110)	0.859	0.979 (0.890, 1.077)	0.663
	Crime safety	0.989 (0.910, 1.076)	0.801	0.926 (0.850, 1.009)	0.079

Abbreviations: OR, odds ratio; CI, confidence interval.

For all the respondents, odds ratios were calculated after adjustment for age, gender, location of the community, physical activity knowledge score, employment status, BMI, marital status, education, and hypertension.

For male and female respondents, odds ratios were calculated after adjusting for age, location of community, physical activity knowledge score, employment status, BMI, marital status, education, and hypertension.

For working and retired individuals, odds ratios were calculated after adjustment for age, gender location of community, physical activity knowledge score, BMI, marital status, education, and hypertension.

For the respective categories, an active walker was defined as a respondent who reported walking for recreation or transportation >90 min/week.

## Discussion

The present study revealed that 13.7% of the overall participants were physical inactive. This prevalence of the physical inactivity was lower compared with several other countries, where 36.1% of males and 37.5% of females were physical inactive in France [26], and the prevalence of physical inactivity was 54.9% in Colombia [27]. The distribution of physical activity differed significantly by gender, age, and employment status. This is consistent with other studies performed in China [5], [6], but inconsistent with many other countries [28]. Interestingly, the distribution of physical activity level reported by Cambodian Americans was 12% low, 40% moderate, and 48% high levels, which is similar to our study [29]. It was suggested that race might be associated with physical activity. Although the prevalence of physical inactivity among Chinese participants in our study was modest, an increasing physical inactivity prevalence among Chinese adults was noted. A WHO worldwide 51-country survey conducted in 2002−2003 revealed that the prevalence of physical inactivity increased significantly among Chinese males [28]. In contrast, the prevalence of physical inactivity in Chinese females with passing time was similar [28]. Some studies attributed the increase in physical inactivity to increased Chinese car ownership, which increased from 0.5−13.1% between 2000 and 2010 [16].

Our study revealed a significant association between perceived accessibility to services and walking for both recreation and transportation in general residents. These results were generally consistent with previous studies performed in Chinese populations [9], [10], [17], [30]. Studies in Hong Kong and mainland China demonstrated that the neighborhood surroundings/aesthetics were not associated with walking for recreation or transportation among general residents [17], [31]. However, the results from our study and that performed Hong Kong are inconsistent with the findings of reports from Western countries [32], [33]. This suggests that a high population density and urban design might influence the association between aesthetics and walking. There was no association between traffic safety and both types of walking in our study. Consistent with this, a neighborhood environment study in 11 countries also reported that high walkability was more important than safety [34]. Another study conducted in China found similar results [31]. It was noteworthy that crime safety was not associated with either type of walking in the present study. Some previous studies reported associations between crime safety and physical activity, with gender-specific differences, in developing countries such as Nigeria and Brazil [35], [36]. Although gender-specific associations between crime safety and walking were examined in our study, no significant associations were identified. However, our observations are consistent with several studies that found no significant association between crime safety and physical activity [37]. This may be related to a safe perception in the living environment as a whole. Because Shanghai is generally perceived to be safe, differences in the perception of crime safety may be insufficient to demonstrate any potential associations.

The associations between environmental variables and walking might differ depending on the participants’ purpose for walking. For males, neighborhood surroundings/aesthetics were significantly negatively associated with walking for recreation, which was inconsistent with previous studies [31], [38], [39]. One reason to explain these findings could be that individuals with a higher income in Shanghai were more likely to live in better neighborhood surroundings, but were physically inactive due to busy social or work schedules [6]. For females, traffic safety was significantly positively associated only with walking for recreation, which was not found in a previous study of elderly Japanese individuals [39]. It was suggested that, as a daily necessary behavior, walking for transportation was less likely to be affected by other factors compared with walking for recreation in Chinese females. In working individuals, there were no associations between environmental variables and either type of walking. For retired participants, accessibility to services was significantly positively associated only with walking for transportation. This may be because retired Chinese individuals’ value walking as a key contributor to health, regardless of their neighborhood environment [40]. Because transportation was not necessary for retired participants who had sufficient time to plan their lives, accessibility to services might influence their decision of whether to walk. Therefore environmental factors may not play an important role in promoting leisure walking in retired individuals.

The association between environmental variables and walking also differed with the characteristics of the participants. Gender differences were observed in the associations between environment and walking in the present study. Although the associations between the environment and walking for recreation were observed in both males and females, the specific environmental factor usually differed. Walking for recreation was significantly associated with accessibility to services and neighborhood surroundings/aesthetics in males, but was associated only with traffic safety in females. Gender-specific analyses also revealed no significant associations between the environment and walking for transportation in females but significant associations in males. Although some previous studies also identified gender-specific associations between the environment and walking, they demonstrated that crime safety, traffic safety, and aesthetics were significant correlates among males, whereas access to shops and exercise facilities were important among females [39]. The main reason for these differences could be that economic status plays a more important role in choice of travel mode in Chinese females compared with males [41].

The associations between the environmental variables and walking for recreation were similar between working and retired individuals. The present study showed that no environmental variables were associated with walking for recreation or transportation in working participants, which was similar to the results of two previous studies [31], [42]. A qualitative study of exercise behavior in China reported that working individuals claimed that lack of time and consciousness of exercise were the most important contributing factors, whereas young people preferred doing other kinds of sports rather than walking [40]. Another study in China found that the main influencing factors in working individuals for physical activity were age and education level [43]. In retired individuals, two studies conducted in Japan found that crime safety and traffic safety were significant correlates among older males [39]. However, inconsistent results were found in our study, since accessibility to services was the only environmental variable associated with walking for transportation. A qualitative study of older adults also reported that accessibility to services (including shops, services, public transit, and connectivity) was important for improving walking [44].

In summary, accessibility to services was important for walking for both recreation and transportation, and traffic safety was also an important factor in walking for transportation in females. However, the neighborhood environment was not an appropriate target for promoting walking in working individuals; therefore other interventions should be considered to promote physical activity in the working population. However, additional studies focusing on physical activity in working individuals are needed.

There are some limitations to our study. First, the direction of causality could not be addressed due to the cross-sectional study design. Second, both walking behaviors and perceived environmental measures were based on self-reporting, even though the measures used have been validated [21], [24], [25]. In spite of these limitations, this study provides novel evidence describing the relationship between physical activity and the environment in China, and will be helpful to understand the environmental correlates of the most common physical activity in Chinese individuals.

## Conclusion

The association between the neighborhood environment and walking was different depending on the purpose for walking and the characteristics of the participants. The findings from our study suggest that interventions to promote walking of community residents by improving neighborhood environment, particularly accessibility to services such as building more stores, parks and public transit facilities should be considered. However, the neighborhood environment was not an appropriate intervention to promote walking in the working population, and therefore other interventions should be adopted to promote physical activity in these individuals.
